# Sexual Orientation–Related Differences in Alcohol Use and Suicide Death

**DOI:** 10.1001/jamanetworkopen.2025.54680

**Published:** 2026-01-20

**Authors:** Sarah McKetta, Landon D. Hughes, Avery M. Anderson, Ran Barzilay, Banapsha Rahman, Kirsty A. Clark

**Affiliations:** 1 Department of Epidemiology, Columbia University Mailman School of Public Health, New York, New York; 2 Department of Population Medicine, Harvard Pilgrim Healthcare Institute, Boston, Massachusetts; 3 Harvard Medical School, Boston, Massachusetts; 4 University of Colorado Anschutz Medical Campus, Aurora; 5 Department of Child and Adolescent Psychiatry and Behavioral Sciences, Children’s Hospital of Philadelphia, Philadelphia, Pennsylvania; 6 Department of Psychiatry, University of Pennsylvania Perelman School of Medicine, Philadelphia; 7 Department of Medicine, Health & Society, Vanderbilt University, Nashville, Tennessee

## Abstract

This cross-sectional study examines the role of any alcohol consumption, intoxication, and alcohol-involved crisis in the suicide death of lesbian, gay, and bisexual decedents compared with heterosexual decedents.

## Introduction

Alcohol use is a factor in suicide. Meta-analyses of prospective studies have shown a 94% higher suicide risk ratio among people who drink alcohol.^1^ Not only is alcohol use associated with increased suicidal thoughts, but its disinhibiting effects increase the likelihood of suicide attempts.^1^

Lesbian, gay, and bisexual (LGB) people in the US are twice as likely to die by suicide as heterosexual people.^2^ They also consume more alcohol.^3^ LGB women, particularly, disproportionately exhibit multiple consumption patterns (eg, binge drinking, daily drinking).^3,4^ LGB suicide and alcohol use inequities are associated with interpersonal discrimination and discriminatory policies.^5^ We aimed to ascertain whether alcohol consumption is a more prevalent precursor to suicide mortality among LGB relative to heterosexual people. This information would inform whether addressing alcohol use is an effective strategy for reducing LGB suicide inequities.

## Methods

We examined alcohol use–related suicide data in the serial cross-sectional National Violent Death Reporting System (NVDRS) from 2013 to 2021. The Harvard Pilgrim Health Care Institute IRB deemed this cross-sectional study exempt from ethics review and informed consent because deidentified surveillance data were used. We followed the STROBE reporting guideline.

Eligible decedents died by suicide (excluding murder-suicide) with known age and sex. We identified LGB or heterosexual orientation using 2 validated strategies^6^: NVDRS-recorded sexual orientation from law enforcement and coroner/medical examiner (LECME) reports and systematic keyword search (eMethods in Supplement 1). LECME retrospectively collected 3 alcohol use measures. For decedents tested for blood alcohol content (BAC), we examined both presence of any BAC above a trace amount (0.01 g/dL) and BAC at the legal intoxication level (≥0.08 g/dL). We additionally created a binary variable flagging alcohol involvement obtained from LECME reports, indicating whether alcohol use was (1) suspected to play a role in the suicide (collected beginning 2016) and/or (2) deemed a “crisis” (ie, recent or acute precipitating factor in the suicide).

We estimated risk ratios (RRs) between LGB orientation and alcohol antecedents (any BAC, intoxication, alcohol involvement) in the full sample and stratified by sex. We used log-binomial distributions, adjusting for age categories (<21, 21-29, 30-39, 40-49, ≥50 years).

Two-sided *P* < .05 indicated statistical significance. Analysis was performed July 2025 using Stata 14.1 (StataCorp).

## Results

The sample included 218 601 decedents (mean [SD] age, 47 [19] years; 169 811 men [77.7%]), including 3425 LGB and 215 176 heterosexual suicides. Of these decedents, 120 590 (55.2%) received BAC testing (Table).

**Table.** Distribution of Alcohol Outcomes Among Eligible Decedents in the 2013-2021 NVDRS, by Sexual Orientation and Sex

Outcome	LGB orientation, No./total No. (%)			Heterosexual, No./total No. (%)		
	Total	Men only	Women only	Total	Men only	Women only
Alcohol involvement^a^	652/3425 (19.0)	367/2048 (17.9)	285/1377 (20.7)	34 777/215 176 (16.2)	27 781/167 763 (16.6)	6996/47 413 (14.8)
Any BAC^b^	906/2107 (43.0)	523/1243 (42.1)	383/864 (44.3)	51 083/118 483 (43.1)	40 041/90 477 (44.3)	11 042/28 006 (39.4)
Intoxication^b^^,^^c^	677/2107 (32.1)	387/1243 (31.1)	290/864 (33.6)	38 623/118 483 (32.6)	30 452/90 477 (33.7)	8171/28 006 (29.2)

Abbreviations: BAC, blood alcohol content; LGB, lesbian, gay, bisexual; NVDRS, National Violent Death Reporting System.

^a^ Alcohol involvement is defined as alcohol use suspected in the hours preceding the incident by witnesses or in investigator reports or as alcohol use that caused a crisis prior to suicide (eg, relapse of someone with alcohol use disorder).

^b^ Numbers represent decedents who were tested for alcohol use.

^c^ Intoxication is defined as BAC of 0.08 g/dL or higher.

The Table shows alcohol use measures by sexual orientation and sex. We saw no marginal association for any BAC (age-adjusted RR [ARR], 1.00 [95% CI, 0.96-1.05]) or intoxication (ARR, 0.99 [95% CI, 0.93-1.05]). There was increased risk, however, for alcohol involvement (ARR, 1.13 [95% CI, 1.06-1.22]).

Stratified by sex, LGB women had increased risks for all alcohol antecedents, but men did not (Figure). Compared with heterosexual women, LGB women had an age-adjusted increased risk of 15% (95% CI, 7%-24%) for any BAC, 17% (95% CI, 7%-28%) for intoxication, and 38% (95% CI, 25%-54%) for alcohol involvement. Tests for interaction for any BAC (χ^2^ = 13.81; *P* = .001), intoxication (χ^2^ = 10.50; *P* = .005), and alcohol involvement (χ^2^ = 38.52; *P* < .001) confirmed differences in associations across LGB orientation and sex.

**Figure.**
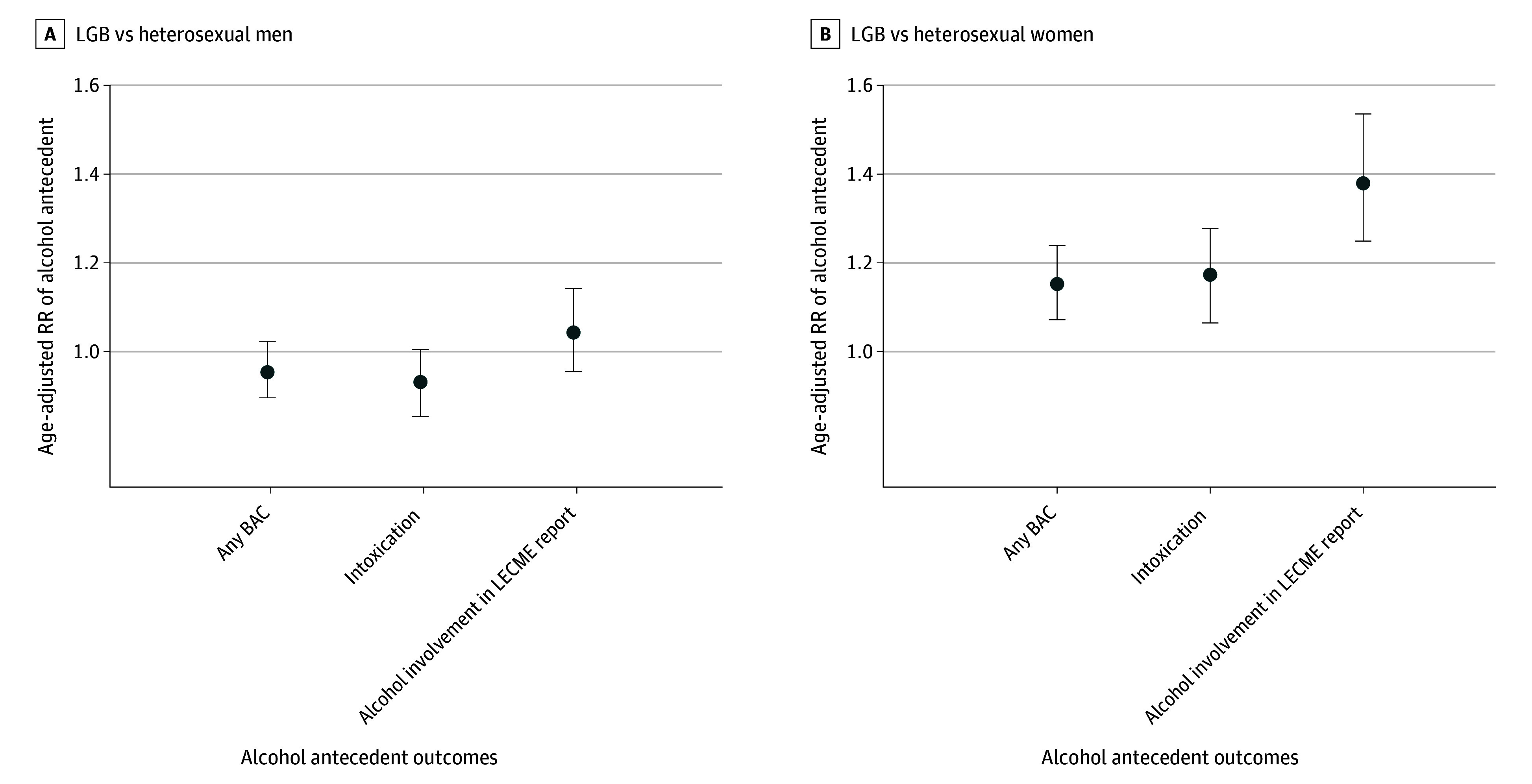
Risk Ratio (RR) of Alcohol Antecedents of Suicide Among Decedents in the 2013 to 2021 National Violent Death Reporting System, Stratified by Lesbian, Gay, Bisexual (LGB) Orientation and Sex Intoxication is defined as blood alcohol content (BAC) of 0.08 g/dL or higher. LECME indicates law enforcement and coroner/medical examiner. Error bars represent 95% CIs.

## Discussion

LGB women’s suicides were more likely to involve alcohol use than heterosexual women’s suicides. This finding is consistent with population-level alcohol use inequities related to sexual orientation, where LGB women have approximately twice the risk of binge drinking as heterosexual women, but LGB and heterosexual men report similar binge drinking prevalences.^4^

This study was limited to cisgender decedents, although research shows transgender people have elevated risks of suicide and alcohol use.^2^ Additionally, keyword search methods for LGB identity in the NVDRS may misclassify some LGB decedents as heterosexual due to limited sexual orientation information, which would bias results toward the null. Our findings underscore the need for further research explicating LGB women’s alcohol use as an intervenable target for suicide prevention.

## Notes

Supplement 1. **eMethods.** Identification of Sexual Orientation in National Violent Death Reporting System

Supplement 2.
Data Sharing Statement
