# Supplementary material for: Sexual Orientation–Related Differences in Alcohol Use and Suicide Death
Source: JAMA Netw Open. 2026 Jan 20;9(1):e2554680. doi: 10.1001/jamanetworkopen.2025.54680 (PMC12820737; doi:10.1001/jamanetworkopen.2025.54680)
Supplement: Supplement 1. — eMethods. Identification of Sexual Orientation in National Violent Death Reporting System [file jamanetwopen-e2554680-s001.pdf]

Supplemental Online Content

McKetta S, Hughes LD, Anderson AM, Barzilay R, Rahman B, Clark KA. Sexual orientation–related differences in alcohol use and suicide death. *JAMA Netw Open*. 2026;9(1):e2554680. doi:10.1001/jamanetworkopen.2025.54680

eMethods. Identification of Sexual Orientation in National Violent Death Reporting System

This supplemental material has been provided by the authors to give readers additional information about their work.

© 2026 McKetta S et al. *JAMA Network Open*

eMethods. Identification of sexual orientation in National Violent Death Reporting System

We identified sexual orientation using the NVDRS coded information on two variables, that is those who were either identified as (1) having partner of the same sex, (2) identified as being lesbian, gay, or bisexual. Each NVDRS record includes 2 narratives summarizing the coroner or medical examiner records and law enforcement reports describing suicide antecedents as reported by the decedent's family or friends; the decedent's diary, social media, and text or email messages; and any suicide note. Using this information, law enforcement and coroner/medical examiners (LECMEs) are trained to demarcate on their reports whether the decedent self-identified as heterosexual, gay, lesbian, or bisexual based on interviews of friends, family, or acquaintances.

Additionally, we adapted and expanded prior approaches[6] to systematically review LECME narratives for sexual orientation information through multistep development of a search algorithm. This algorithm was developed using query terms (e.g., lesbian, gay, bisexual) to search the narratives; then a random sample of decedents was identified from this initial search and reviewed to further refine the keywords and criteria (e.g., "his boyfriend"). Those identified as a sexual minority were coded as a sexual minority, while those we could not identify their sexual orientation were coded as "presumably heterosexual." In development of this algorithm, multiple reviewers double-coded 50 narratives and had 98% agreement (k=0.96).[6]

© 2026 McKetta S et al. *JAMA Network Open*
