# Supplementary material for: Sexual Orientation–Related Differences in Alcohol Use and Suicide Death
Source: JAMA Netw Open. 2026 Jan 20;9(1):e2554680. doi: 10.1001/jamanetworkopen.2025.54680 (PMC12820737; doi:10.1001/jamanetworkopen.2025.54680)
Supplement: Supplement 2. — Data Sharing Statement [file jamanetwopen-e2554680-s002.pdf]

Data Sharing Statement

McKetta. Sexual Orientation–Related Differences in Alcohol Use and Suicide Death. *JAMA Netw Open*. Published January 20, 2026. doi:10.1001/jamanetworkopen.2025.54680

Data
Data available: No

Additional Information
Explanation for why data not available: We do not have permission to share the data, but they are available by request to NVDRS RAD
